# Effect of the angiotensin II receptor antagonist olmesartan on morning home blood pressure in hypertension: HONEST Study at 16 weeks

**DOI:** 10.1038/jhh.2013.68

**Published:** 2013-07-18

**Authors:** K Kario, I Saito, T Kushiro, S Teramukai, Y Ishikawa, K Hiramatsu, F Kobayashi, K Shimada

**Affiliations:** 1 Division of Cardiovascular Medicine, Department of Medicine, Jichi Medical University School of Medicine, Shimotsuke, Japan; 2 Keio University Health Center, Yokohama, Japan; 3 Health Planning Center, Nihon University School of Medicine, Tokyo, Japan; 4 Innovative Clinical Research Center, Kanazawa University, Kanazawa, Japan; 5 Daiichi Sankyo Co., Ltd., Tokyo, Japan; 6 Shin-Oyama City Hospital, Oyama, Japan

**Keywords:** angiotensin receptor antagonists, clinic blood pressure, home blood pressure monitoring, morning hypertension, olmesartan medoxomil

## Abstract

Morning home blood pressure (BP) levels are more closely associated with cardiovascular risk than clinic BP levels. However, control of morning home BP has been worse than that of clinic BP in clinical practice. We examined the effects of olmesartan-based treatment using data (*n*=21 341) from the first 16 weeks of the Home BP measurement with Olmesartan Naive patients to Establish Standard Target blood pressure (HONEST) study, a prospective observational study for olmesartan-naive patients with essential hypertension. After 16-week olmesartan-based treatment, the clinic and morning home systolic BP (SBP) lowered from 151.6±16.4 and 153.6±19.0 mm Hg to 135.0±13.7 and 135.5±13.7 mm Hg, respectively (*P*<0.0001). The achievement percentage of target morning home SBP (<135 mm Hg) in all patients, those with diabetes mellitus (DM), and those with chronic kidney disease (CKD) increased from 13.5, 16.4 and 17.2% to 50.8, 47.9 and 48.8%, respectively, and the proportion of patients with well-controlled hypertension (clinic SBP<140 mm Hg and morning home SBP<135 mm Hg) increased from 7.9, 9.2 and 10.2% to 38.9, 34.5 and 36.3%, respectively. After 16-week olmesartan-based treatment, the proportion of patients with masked and white coat hypertension changed from 11.8 to 24.2% and 5.6 to 11.9%. In conclusion, both clinic and morning home BP in all, DM and CKD patients improved with 16-week olmesartan-based treatment in the ‘real world', and the results showed a sustained 24-hour BP-lowering effect of olmesartan. Decrease in clinic and home BP resulted in an increased rate of masked and white coat hypertension, and further management is needed in those patients.

## Introduction

Hypertension is a well-known risk factor for cardiovascular disease. To reduce this risk, strict control of blood pressure (BP) is recommended by various guidelines for hypertension treatment.^1, 2, 3^

However, in the Jichi Morning Hypertension Research Study (the J-MORE study), a cross-sectional study that showed standard antihypertensive treatment under conditions of daily clinical practice in Japan, clinic BP was controlled in only 42% of the patients, and moreover, 61% of these patients had masked hypertension because of high morning BP.^4^

Cardiovascular events tend to occur most frequently in the morning along with a peak of ambulatory BP,^5^ and the morning systolic BP (SBP) is the strongest independent predictor for stroke among clinic, 24-hour, awake, sleep, evening, pre-awake and morning BPs.^6^ Therefore, antihypertensive treatment for morning hypertension is likely to offer greater benefit in preventing cardiovascular events.

Home BP monitoring has several benefits. Compared with clinic BP, home BP measurements provide a more accurate prognosis for survival.^7, 8, 9^ Home BP measurements can also be combined with clinic BP measurements to differentiate patients with masked hypertension, white coat hypertension, poor-controlled hypertension and well-controlled hypertension. It is important to identify patients in these groups, because masked hypertension has a cardiovascular risk at least equal to that of poor-controlled hypertension.^10, 11^ Furthermore, white coat hypertension can be a risk factor for stroke in the long term, according to a pooled analysis of ambulatory blood pressure monitoring (ABPM) data from an international collaborative study.^12^

Home BP is appropriate for evaluating the sustained 24-hour BP-lowering effect of an antihypertensive drug, whereas clinic BP is not appropriate.^13^

The Home BP measurement with Olmesartan Naive patients to Establish Standard Target blood pressure (HONEST) study is a large-scale prospective observational study following>20 000 patients receiving olmesartan-based antihypertensive treatment for 2 years; time from start of treatment to first occurrence of cardiovascular events is the primary endpoint.^14^

In the present analysis, we used measurements of morning home BP at the first measurement and clinic BP from the first 16 weeks of the HONEST study to evaluate the antihypertensive efficacy of olmesartan and the sustained 24-hour BP-lowering effect in patients with diabetes mellitus (DM) and chronic kidney disease (CKD) in addition to the overall patient population. We also investigated the effects of olmesartan on morning hypertension.

## Materials and methods

### Study protocol

This study is a prospective observational study with a 2-year follow-up. The aims and protocol have already been reported.^14^ This study protocol was approved by the Ministry of Health, Labour and Welfare of Japan (MHLW) before study commencement. This study was carried out in medical institutions registered in compliance with Good Post-marketing Study Practice in Japan and internal regulations for clinical studies at each institution. The study is registered at http://www.umin.ac.jp/ctr/index.htm under the unique trial number UMIN000002567.

In brief, participants were olmesartan-naive patients with essential hypertension and no history of recent acute cardiovascular events (for example, myocardial infarction, stroke and cardiovascular interventions), and with no planned cardiovascular interventions. Written informed consent was obtained from them at the start of this study. Olmesartan (generally 10 or 20 mg per day) was administered at each participating physician's discretion. The selection of target clinic BPs and home BPs was left to the discretion of the individual physicians. No restriction was placed on prior antihypertensive drug treatment, with the exception of prior use of olmesartan, or on the use of combination antihypertensive drug treatment during the study. The data included patient characteristics (for example, disease history and complications), clinic and home BP, clinical laboratory test values, and the incidence of cardiovascular events and adverse events during the study period. The present analysis used data from the HONEST study for patients who received olmesartan in the first 16 weeks.

### Home BP measurements

The patients who already owned electronic arm-cuff devices based on the cuff-oscillometric method were registered. All such devices available in Japan have been validated and approved by MHLW. At the time of obtaining informed consent, patients were asked to measure home BP twice in the morning and twice at bedtime according to the Japanese Society of Hypertension,^3^ namely, within 1 h of waking in the morning (after urinating, before their dose of antihypertensive agents, before breakfast and after 1–2 min of rest in a sitting position) and at bedtime (after 1–2 min of rest in a sitting position). We analyzed only the first measurement of home BP and pulse rate in the morning at baseline and at 1, 4 and 16 weeks. Home BP at each measurement point was defined as an averaged value over 2 days.

### Definition of control status by clinic and morning home BP

The BP control status of patients was defined based on the European Society of Hypertension guidelines for BP monitoring at home, which state that home BP monitoring can provide information about BP control outside the office, thereby allowing the identification of treated hypertensive patients with white coat hypertension and masked hypertension.^15^

In this report, we defined the control status as the clinic and morning home BP at the first measurement as follows: morning hypertension was defined as morning home SBP⩾135 mm Hg; masked hypertension was defined as clinic SBP<140 mm Hg and morning home SBP⩾135 mm Hg; white coat hypertension was defined as clinic SBP⩾140 mm Hg and morning home SBP<135 mm Hg; poor-controlled hypertension was defined as clinic SBP⩾140 mm Hg and morning home SBP⩾135 mm Hg; and well-controlled hypertension was defined as clinic SBP<140 mm Hg and morning home SBP<135 mm Hg. At baseline, each defined hypertension group included both patients receiving and not receiving the treatment. We have reported the same criteria for the diagnosis and classification of patients, including treated patients, in a previous article regarding the protocol of this study.^14^ We checked the results in the group of untreated hypertensive patients without pre-existing cardiovascular disease (*n*=9888) at baseline.

### Definition of patients with DM and CKD

The patients with DM at baseline were selected from the physician's report. The patients with CKD at baseline, defined as having an estimated glomerular filtration rate (eGFR) <60 ml min^−1^ per 1.73 m^2^, or proteinuria higher than 2+ on the dipstick test, or proteinuria 1+ and renal disease as a complication at study entry, or both. eGFR was calculated using the following formula devised for the Japanese population:^16^ eGFR=194 × age (years)^−0.287^ × SCr^−1.094^ ( × 0.739 in women), where serum creatinine (SCr) levels measured within 12 months before study onset were used. The target BP for hypertensive patients with DM or CKD is currently a topic of debate around the world. In this paper, we considered the targets to be clinic SBP<140 mm Hg and morning home SBP<135 mm Hg.

### Statistical analysis

**Figure 1:**
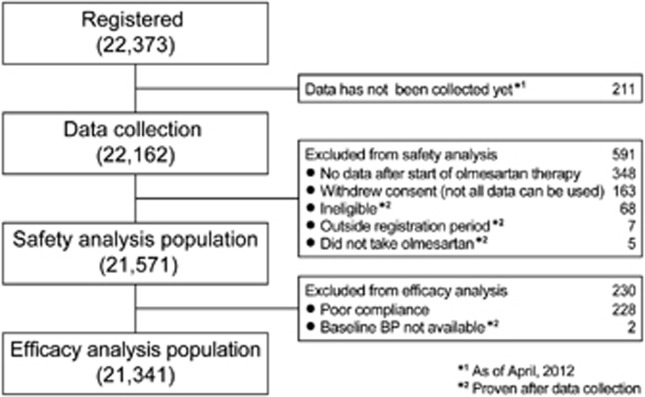
Patient flow in the HONEST study.

The safety analysis population was defined as eligible patients who received olmesartan at least once during the treatment period. The efficacy analysis population was defined as the population excluded from the safety analysis population because of poor compliance with olmesartan, which was reported by the study investigator as ‘almost never taken the study drug' and/or with missing data for BP at baseline (Figure 1). These exclusion criteria were defined in accordance with those of a previous study.^17^

We analyzed evaluable data of BP control in the efficacy analysis population and safety data in the safety analysis population. Continuous variables and categorical variables were expressed as the mean±s.d. and proportion (%), respectively.

For the analysis of changes in SBP, DBP and pulse rate, the Dunnett–Hsu test was used to compare measurements at 1 (for home BP and pulse rate only), 4 and 16 weeks with baseline values to adjust for multiple comparisons. McNemar's test was adapted to evaluate the efficacy of olmesartan based on changes in the distribution of well-controlled patients and the others between baseline and 16 weeks, and 16 weeks of achievement rate of target morning home SBP (<135 mm Hg) and clinic SBP (<140 mm Hg).

A two-sided test was used and *P*<0.05 was considered to be statistically significant. SAS release 9.2 software (SAS Institute, Cary, NC, USA) was used for all statistical analyses. Adverse events considered by the study investigator to be related to olmesartan were classified as adverse drug reactions (ADRs). ADRs were classified based on the preferred term from the Medical Dictionary for Regulatory Activities.

## Results

### Study profile

The number of patients registered and reasons for exclusion of data from statistical analyses are shown in Figure 1. A total of 22 373 patients throughout Japan were registered between October 1, 2009 and September 30, 2010. The dataset in this analysis was used as of April 2012. Data for the first 16 weeks were collected from 22 162 patients. Data from 21 571 and 21 341 patients were used for the analyses of safety and efficacy, respectively.

### Patient characteristics

The baseline characteristics of the 21 341 patients in the efficacy analysis are presented in Table 1. Average age of the patients was 64.8±11.9 years; 50.5% of patients were women; 10.5% had a history of stroke, percutaneous coronary intervention, or myocardial infarction; 50.3% were receiving antihypertensive agents before the start of olmesartan treatment. The prevalence of DM and CKD was 20.4% and 20.1%, respectively.

At baseline, morning home SBP/DBP and pulse rate were 151.6±16.4/87.1±11.8 mm Hg and 70.8±10.0 beats min^−1^, respectively. Clinic SBP/DBP and pulse rate were 153.6±19.0/87.1±13.4 mm Hg and 74.1±11.2 beats min^−1^, respectively.

### Administration status of antihypertensive agents

The administration status of antihypertensive agents is shown in Table 2. Average dose of olmesartan and the number of antihypertensive agents including olmesartan slightly increased from 18.2±7.0 mg per day and 1.5±0.7 at the start of olmesartan treatment to 18.8±8.3 mg per day and 1.6±0.8 at 16 weeks.

### Changes in BP and pulse rate

**Figure 2:**
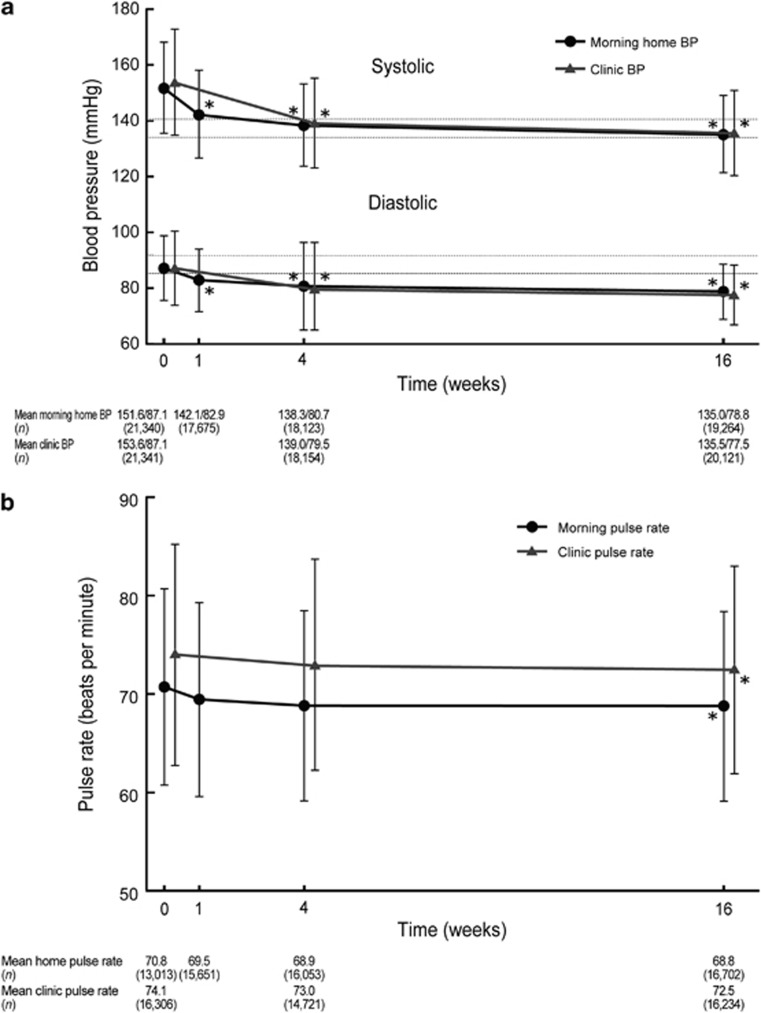
Changes in BP (**a**) and pulse rate (**b**) over 16 weeks of olmesartan treatment. *P<0.0001 (versus just before olmesartan treatment, Dunnett–Hsu test).

Changes in the morning home BP and pulse rate as well as clinic BP and pulse rate throughout the study period are shown in Figure 2. At baseline, morning home SBP/DBP and clinic SBP/DBP were 151.6±16.4/87.1±11.8 mm Hg and 153.6±19.0/87.1±13.4 mm Hg. Morning home SBP/DBP decreased significantly at 1 week, down to 135.0±13.7/78.8±9.9 mm Hg at 16 weeks (*P*<0.0001 for all comparisons). Clinic SBP/DBP had decreased by 4 weeks and was 135.5±15.3/77.5±10.9 mm Hg at 16 weeks (*P*<0.0001 for all comparisons). After 16 weeks of olmesartan-based treatment, morning home pulse rate and clinic pulse rate lowered from 70.8±10.0 and 74.1±11.2 beats min^−1^ to 68.8±9.5 and 72.5±10.5 beats min^−1^, respectively (both *P*<0.0001).

In a group of previously untreated hypertensive patients without pre-existing cardiovascular disease (*n*=9888), morning home SBP/DBP and clinic SBP/DBP decreased significantly from 156.6±15.4/91.0±11.1 and 159.7±17.8/91.8±12.4 mm Hg to 134.8±13.4/79.8±9.5 and 135.6±14.8/78.8±10.4 mm Hg, and morning home pulse rate and clinic pulse rate lowered from 72.0±9.8 and 74.6±10.9 beats min^−1^ to 69.5±9.2 and 72.4±9.9 beats  min^−1^, after 16 weeks of olmesartan-based treatment, respectively (all *P*<0.0001).

### Antihypertensive effects and sustained 24-hour BP-lowering effect of olmesartan

**Figure 3:**
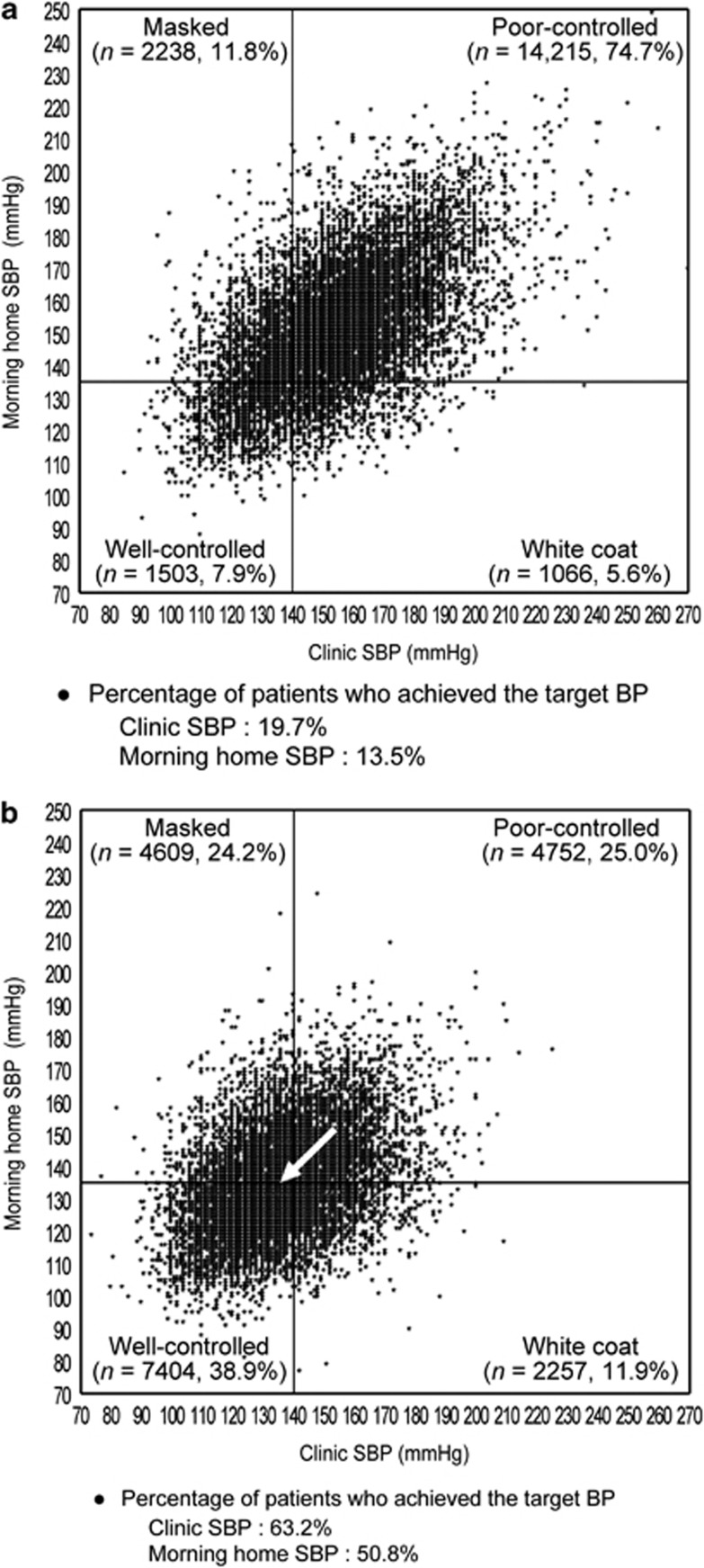
Morning home and clinic systolic blood pressure (SBP) in patients with well-controlled, white coat, masked, and poor-controlled hypertension (**a**) just before and (**b**) after 16 weeks of olmesartan treatment (*n*=19 022). There was a significant difference between just before and after 16 weeks of olmesartan treatment (*P*<0.0001, McNemar's test). The arrow shows the change in average SBP from baseline to 16 weeks.

The distribution of patients across the four groups based on clinic SBP (cut-off value, 140 mm Hg) and morning home SBP (cut-off value, 135 mm Hg) at the start of olmesartan treatment and at 16 weeks is shown in Figure 3. Distribution of well-controlled patients significantly increased at 16 weeks of olmesartan treatment (*P*<0.0001). The proportion of patients who had poor-controlled and well-controlled hypertension changed from 74.7 to 25.0% and from 7.9 to 38.9% between baseline and 16 weeks of olmesartan-based treatment, respectively. As before, the percentage of patients who had masked hypertension and white coat hypertension changed from 11.8 to 24.2% and 5.6 to 11.9%, respectively.

In a group of previously untreated hypertensive patients without pre-existing cardiovascular disease (*n*=8746), the proportion of patients who had well-controlled, poor-controlled, masked and white coat hypertension changed from 2.8, 88.1, 6.1 and 3.0% to 39.8, 25.2, 23.7 and 11.3% after 16 weeks of olmesartan-based treatment.

The proportion of patients who achieved target SBP at baseline and 16 weeks increased from 19.7 to 63.2% for clinic SBP (<140 mm Hg) and from 13.5 to 50.8% for morning home SBP (<135 mm Hg), which meant that the proportion of patients who had morning hypertension decreased from 86.5 to 49.2% (all *P*<0.0001).

In a group of previously untreated hypertensive patients without pre-existing cardiovascular disease (*n*=8746), the proportion of patients who achieved clinic SBP<140 mm Hg, morning home SBP<135 mm Hg and morning hypertension changed from 8.9, 5.8 and 94.2% to 63.5, 51.1 and 48.9% after 16 weeks of olmesartan-based treatment (all *P*<0.0001).

**Figure 4:**
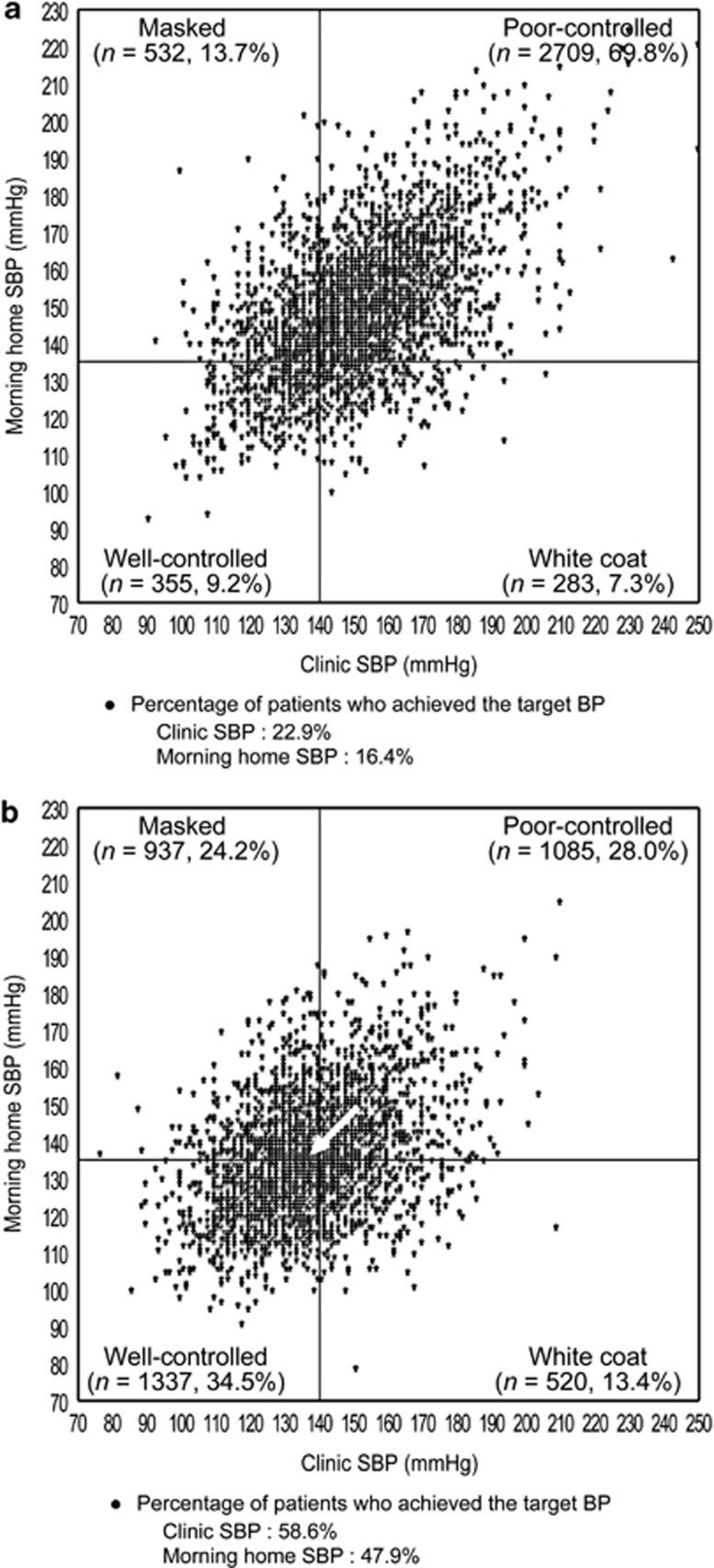
Morning home and clinic SBP in patients with well-controlled, white coat, masked and poor-controlled hypertension (**a**) just before and (**b**) after 16 weeks of olmesartan treatment in a subgroup of patients with diabetes mellitus (*n*=3879). There was a significant difference between just before and after 16 weeks of olmesartan treatment (*P*<0.0001, McNemar's test). The arrow shows the change in average SBP from baseline to 16 weeks.

**Figure 5:**
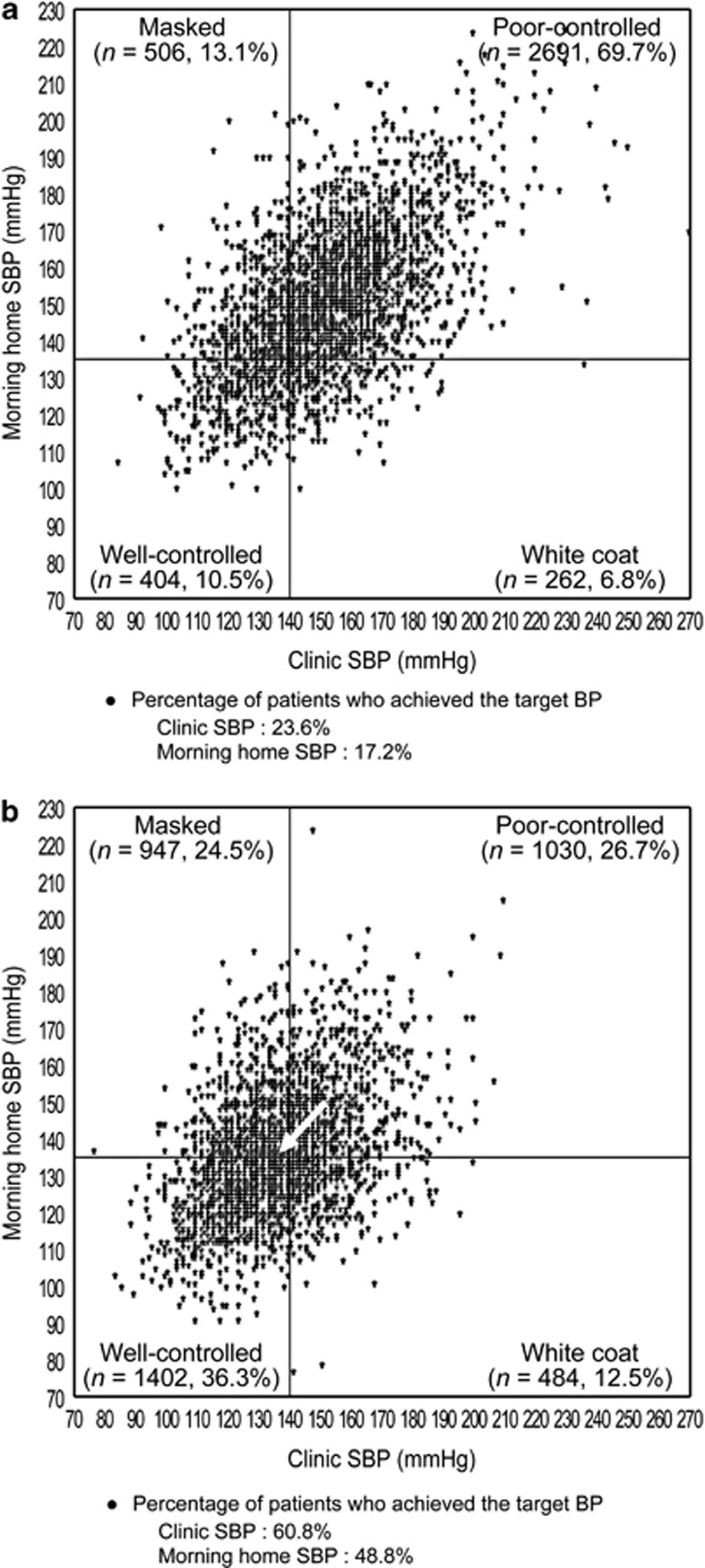
Morning home and clinic SBP in patients with well-controlled, white coat, masked and poor-controlled hypertension (**a**) just before and (**b**) after 16 weeks of olmesartan treatment in a subgroup of patients with chronic kidney disease (*n*=3863). There was a significant difference between just before and after 16 weeks of olmesartan treatment (*P*<0.0001, McNemar's test). The arrow shows the change in average SBP from baseline to 16 weeks.

The distribution of patients across the four groups based on clinic SBP and morning home SBP in patients with DM and CKD is shown in Figures 4 and 5. Distribution of well-controlled patients significantly increased at 16 weeks of olmesartan treatment (both *P*<0.0001). The proportion of patients with DM who had poor-controlled and well-controlled hypertension changed from 69.8 to 28.0% and 9.2 to 34.5%, and that of patients with CKD also changed from 69.7 to 26.7% and 10.5 to 36.3% between baseline and 16 weeks of olmesartan treatment, respectively.

In a group of previously untreated hypertensive patients with DM without pre-existing cardiovascular disease (*n*=1267), the proportion of patients who had well-controlled, poor-controlled, masked and white coat hypertension changed from 3.9, 83.5, 7.3 and 5.4% to 36.5, 26.4, 24.2 and 12.8% after 16 weeks of olmesartan-based treatment. In a group of previously untreated hypertensive patients with CKD without pre-existing cardiovascular disease (*n*=1263), the proportion of patients who had well-controlled, poor-controlled, masked and white coat hypertension changed from 3.2, 87.7, 6.0 and 3.1% to 37.5, 26.8, 23.8 and 12.0% after 16 weeks of olmesartan-based treatment.

The proportion of patients with DM who achieved target SBP at baseline and 16 weeks increased from 22.9 to 58.6% for clinic SBP (<140 mm Hg) and from 16.4 to 47.9% for morning home SBP (<135 mm Hg). Similarly, the proportion of patients with CKD who achieved target SBP increased from 23.6 to 60.8% for clinic SBP and from 17.2 to 48.8% for morning home SBP. So this meant that the proportion of patients with DM who had morning hypertension decreased from 83.6 to 52.1% and that of patients with CKD decreased from 82.8 to 51.2% (all *P*<0.0001).

In a group of previously untreated hypertensive patients with DM without pre-existing cardiovascular disease (*n*=1267), the proportion of patients who achieved clinic SBP<140 mm Hg, morning home SBP<135 mm Hg and morning hypertension changed from 11.1, 9.2 and 90.8% to 60.8, 49.3 and 50.7% after 16 weeks of olmesartan-based treatment (all *P*<0.0001). In a group of previously untreated hypertensive patients with CKD without pre-existing cardiovascular disease (*n*=1263), the proportion of patients who achieved clinic SBP (< 140 mm Hg), morning home SBP (< 135 mm Hg) and morning hypertension changed from 9.2, 6.3 and 93.7% to 61.2, 49.5 and 50.5% after 16 weeks of olmesartan-based treatment (all *P*<0.0001).

### Safety

Of the 21 571 patients whose data were included in the safety analysis, 286 patients (1.33%) experienced ADRs. Major ADRs were dizziness (0.23%), decreased BP (0.10%) and hypotension (0.09%). ADRs associated with excessive BP lowering, which consisted of dizziness, decreased BP, hypotension, dizziness, and postural and orthostatic hypotension, occurred in 0.48% of the patients.

## Discussion

This study is the first to show how the distribution of patients across the four groups (poor-controlled hypertension, masked hypertension, white coat hypertension and well-controlled hypertension by olmesartan-based antihypertensive treatment) changed in patients with DM and CKD in addition to the overall patient population in the ‘real world.' Our findings were essentially the same in both patient groups defined in the present study and in untreated hypertensive patients without pre-existing cardiovascular disease.

Moreover, this is the first study to show a sustained 24-hour BP-lowering effect of olmesartan, evaluated using morning home and clinic BP in the ‘real world' utilizing data from a large number of patients.

### Antihypertensive effects of olmesartan on BP

The clinic BP and morning home BP had decreased significantly to about 135/85 mm Hg, the normal level of the morning home BP, at 16 weeks of olmesartan-based treatment.

The results show that olmesartan has a sustained 24-hour BP-lowering effect, because of similar efficacy on the morning home BP compared with the clinic BP. This is supported by the results from a meta-analysis of double-blind clinical trials guided by the ABPM^18^ and the results of a randomized clinical trial, which show that olmesartan administered in the morning is as effective as olmesartan administered in the evening in decreasing morning home BP, office BP and urinary albumin-to-creatinine ratio.^19^

The proportion of patients with morning hypertension was reduced from 86.5 to 49.2% after olmesartan treatment. Morning hypertension is classified into the ‘nocturnal hypertension' type and the ‘morning surge' type.^20, 21^ Hypertensive patients on standard antihypertensive treatment often have morning hypertension^4^ of the nocturnal hypertension type, because the effect of most antihypertensive drugs does not last for 24 h. The renin–angiotensin system activation during sleep and early morning periods is considered to contribute to morning hypertension of the morning surge type hypertension.^22^ The mean levels of the morning home BP were similar to the mean levels of the clinic BP after olmesartan-based treatment for 16 weeks. Therefore, it suggests that olmesartan improves both types of morning hypertension and that olmesartan-based treatment is effective for control of morning hypertension.

In this study, more patients had masked hypertension or white coat hypertension at 16 weeks (24.2% and 11.9%, respectively) than at baseline. That is, masked hypertension and white coat hypertension can be considered to be a sign of being on the way to achieving well-controlled hypertension, but that the antihypertensive effects are insufficient. Generally, antihypertensive treatment for poor-controlled hypertension results in not only well-controlled hypertension, but also masked hypertension and white coat hypertension.^17, 23^ However, this important fact is likely to be easily missed in daily clinical practice. This fact shows that it is important to keep treating hypertensive patients guided by both clinic BP and home BP through antihypertensive treatment in order to control both BPs. Several reports showed that under antihypertensive treatment the proportion of patients with masked hypertension is 11–33%,^24, 25, 26^ and that of patients with white coat hypertension is 12–19%,^23, 25, 27^ although the definitions of each condition vary depending on the studies.

Morning home BP decreased significantly after the first week of treatment. Saito *et al.*^28^ have reported that olmesartan is safe and effective in reducing clinic BP after 1 week of treatment.

In this study, ADRs and ADRs associated with excessive BP lowering occurred in 1.33% and 0.48% of the patients, respectively. It suggests that olmesartan is safe and that the antihypertensive effect of olmesartan lasts until morning in the first week of treatment. Early reduction in BP at the first week could improve patient adherence, thereby enhancing reduction of cardiovascular risk.^29, 30^

### Effects of olmesartan on pulse rate

Both clinic pulse rate and morning home pulse rate decreased significantly after 16 weeks of olmesartan-based treatment. Olmesartan might exert an inhibitory action on the sympathetic nervous system activity.

### Antihypertensive effects of olmesartan in patients with DM and CKD

The proportion of poor-controlled hypertension was reduced and the proportion of well-controlled hypertension was increased in the DM and CKD patients between baseline and 16 weeks of olmesartan-based treatment. It suggests that olmesartan-based treatment is effective for the control of morning home SBP and clinic SBP in high-risk patients such as patients with DM and CKD. Moreover, it also suggests that higher doses of olmesartan or addition of other antihypertensive drugs is often needed to control both BPs.

### Well-controlled hypertension achieved by olmesartan-based treatment

There was no control group in this study, so we tried to compare the BP control status with four similar cohort studies (the SHEAF study,^10^ the J-HOME study,^24^ the J-MORE study,^4, 31^ and the At-HOME study^17^) conducted in treated hypertensive patients as historical controls. The proportions of patients with well-controlled (clinic BP<140/90 mm Hg and morning home BP<135/85 mm Hg) hypertension in the SHEAF study (*n*=4928), the J-HOME study (*n*=3400) and this study (*n*=19 019) were 13.9, 19.1 and 33.1% (data not shown), respectively. The proportions of patients with well-controlled (clinic SBP<140 mm Hg and morning home SBP<135 mm Hg) hypertension in the J-MORE study (*n*=969), the At-HOME study (*n*=4074) and this study (*n*=19 022) were 21.1, 32.2 and 38.9%, respectively. Although direct comparison with these studies is somewhat problematic, the proportions of patients with well-controlled hypertension in this study were higher than in those of other studies. The following two factors might have contributed to the higher proportion of patients with well-controlled hypertension in this study in comparison with previous studies, including a recent similarly designed study.^17^ First, hypertension treatment guidelines^1, 2, 3^ that promote stricter BP control were released after the other studies ended. Second, the more potent antihypertensive effect and sustained 24-hour BP-lowering effect of olmesartan,^32–34^ as well as its pleiotropic effects,^35, 36^ could have had a favorable influence.

The study included 249 (2.8%) of the previously untreated patients without pre-existing cardiovascular disease in a group with well-controlled hypertension at baseline. Of these patients, 130 were without DM or CKD and did not meet the criteria for hypertension (that is, clinic DBP⩾90 mm Hg or morning home DBP⩾85 mm Hg). Although these patients were untreated at baseline, they were being followed up because of their history of hypertension. Although their BP measurements on the day of starting olmesartan-based therapy were normal, drug treatment was indicated by previous high BP measurements.

### Study limitations

There are some limitations in the current study. First, the design of the HONEST study was to represent the ‘real world' of clinical practice, and consequently patients were not blinded to treatment and there was no control group. However, the results of the present study according to sustained 24-hour BP-lowering effect of olmesartan were similar to those of the previous double-blind clinical trials.^32, 33, 34^ Second, the definitions of BP control status that we used for both treated and untreated hypertensive patients in the present study are inconsistent with the stricter definitions used in the previous studies involving general populations. Finally, because the present study lacks data for daytime BP and nocturnal BP, there was the potential for missing stress-induced hypertension and nocturnal hypertension. This may lead to underestimation of the prevalence of masked hypertension and overestimation of the prevalence of white coat hypertension. However, regarding diagnostic accuracy, Nasothimiou *et al.*^37^ have reported that home BP seems to be a reliable alternative to ambulatory BP in the diagnosis of hypertension and the detection of white coat and masked hypertension in both untreated and treated subjects. Nevertheless, the evaluations done in this study, including the four definitions of BP control status and graphical analyses grouping data using patients' BP control status, are a simple and useful method for both physicians and patients when evaluating BP control under daily clinical practice, because the current BP status and changes in BP status are easily visualized. The relation between the diagnoses and classifications used in this study and the occurrence of cardiovascular events will be examined when the HONEST study is complete, and will further clarify the clinical significance of this method of evaluation.


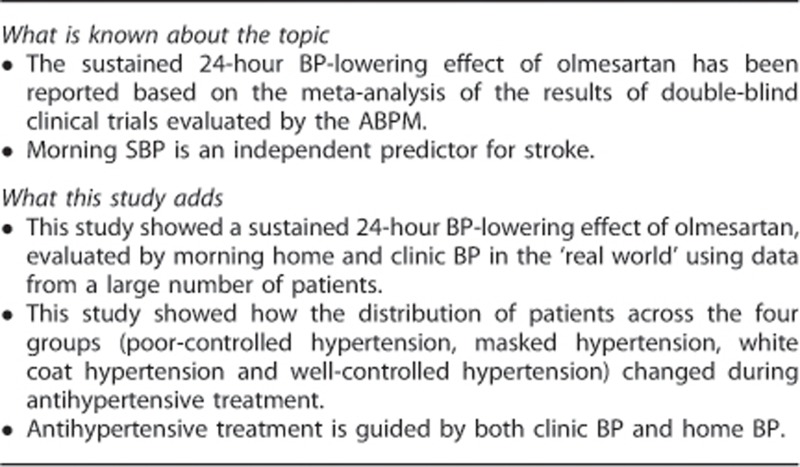


## Figures and Tables

**Table 1:** Baseline characteristics of patients whose data were used in the efficacy analysis (*n*=21 341)

	*No. of patients (%) or mean±s.d.*
Women	10 784 (50.5)
Age (years)	64.8±11.9
Body mass index (kg m^−2^)	24.31±3.70
	
*Disease history*	
Cerebral or cardiovascular disease	2242 (10.5)
Cerebrovascular disease	1416 (6.6)
Cardiovascular disease	966 (4.5)
Previous antihypertensive agents	10 732 (50.3)
Calcium channel blocker	7690 (36.0)
Angiotensin II receptor blocker	4535 (21.3)
β-Blocker	1336 (6.3)
Diuretic	1230 (5.8)
Angiotensin-converting enzyme inhibitor	780 (3.7)
α-Blocker	454 (2.1)
Other	90 (0.4)
	
*Complications*	
Dyslipidemia	9484 (44.4)
Diabetes mellitus	4364 (20.4)
Cardiac disease	1983 (9.3)
Chronic kidney disease	4284 (20.1)
	
*Morning home BP measurements*	
Systolic BP (mm Hg) (the first time)	151.6±16.4
Diastolic BP (mm Hg) (the first time)	87.1±11.8
Pulse rate (beats min^−1^) (the first time)	70.8±10.0
	
*Timing of morning home BP measurement*	
Before taking antihypertensive agents	19 497 (91.4)
After taking antihypertensive agents	535 (2.5)
Unknown	1309 (6.1)
	
*Clinic measurements*	
Systolic BP (mm Hg)	153.6±19.0
Diastolic BP (mm Hg)	87.1±13.4
Pulse rate (beats min^−1^)	74.1±11.2

Abbreviation: BP, blood pressure.

**Table 2:** Administration status of antihypertensive agents^a^

	*At the start of olmesartan therapy (baseline) (*n*=21 341)*	*16 weeks (*n*=21 341)*
Dose of olmesartan, mean±s.d. (mg per day)	18.2±7.0	18.8±8.3
0 (discontinuation)	0 (0.0)	512 (2.4)
0<−5 (mainly 5)	502 (2.4)	489 (2.3)
5<−10 (mainly 10)	5450 (25.5)	4455 (20.9)
10<−20 (mainly 20)	14 193 (66.5)	13 982 (65.5)
20<−40 (mainly 40)	1196 (5.6)	1903 (8.9)
Receiving concomitant antihypertensive agents	8280 (38.8)	9588 (44.9)
Calcium channel blocker	7245 (33.9)	8389 (39.3)
β-Blocker	1276 (6.0)	1386 (6.5)
Diuretic	961 (4.5)	1403 (6.6)
α-Blocker	436 (2.0)	509 (2.4)
Angiotensin-converting enzyme inhibitor	309 (1.4)	309 (1.4)
Angiotensin II receptor blocker	160 (0.7)	161 (0.8)
Other	74 (0.3)	112 (0.5)
No. of antihypertensive drugs (including olmesartan)	1.5±0.7	1.6±0.8

a Values are *n* (%) unless otherwise specified.

## Notes

Dr Kario, Dr Saito, Dr Kushiro, Dr Teramukai and Dr Shimada received honoraria from Daiichi Sankyo Co., Ltd. Mr Ishikawa, Mr Hiramatsu and Mr Kobayashi are employees of Daiichi Sankyo Co., Ltd.
